# Effects of herbicides on non-target plant species diversity and the community composition of fallow fields in northern China

**DOI:** 10.1038/s41598-020-67025-2

**Published:** 2020-06-19

**Authors:** Yue Qi, Junsheng Li, Xiao Guan, Bing Yan, Gang Fu, Jing He, Leshan Du, Caiyun Zhao, Dun Zhang

**Affiliations:** 1 State Environment Protection Key Laboratory of Regional Eco-processand Function Assessment, Chinese Research Academy of Environmental Sciences, Beijing, 100012 China; 2 State Key Laboratory of Environmental Criteria and Risk Assessment, Beijing, 100012 China

**Keywords:** Ecology, Environmental sciences

## Abstract

Despite the important ecological and agricultural production value of fallow field vegetation in agricultural landscapes, it is often affected by herbicide drift and runoff from neighboring sprayed fields. However, little is known about the impact of herbicides on the non-target plant community of fallow fields. In this study, the plant community of fallow fields was investigated following annual sublethal exposure to atrazine or tribenuron-methyl by a 3-year (2014–2016) randomized block field study. The two herbicides both changed the species composition, reduced the number of plant species and the relative frequencies of some plants, and significantly reduced the Margalef species richness index and Shannon’s diversity index of the plant community in the fallow field. The effects of the two herbicides on species number and community composition were not consistent. The effects of herbicide doses less than the recommended field application concentration (RFAC) on the plant community composition and community diversity of the fallow field were not lower than the effects of the RFAC of the herbicides. Indeed, doses less than the RFAC had an even greater impact on the community diversity than the RFAC of the herbicides. As the number of years of herbicide application increased, the effects of the herbicides on the plant community diversity did not increase compared to the effects of the blank control, and the herbicides did not change the functional composition of the plant communities in the fallow field. Our results suggest that the ecological risks of herbicides, even at low concentrations, on non-target wild plant communities in agricultural landscapes should not be neglected in the development of practical plant diversity conservation strategies.

## Introduction

The community diversity and the functional diversity of wild plants in agroecosystems, especially in field edges, fallow fields, and other semi-natural habitats, provide us with ecosystem and agricultural production services^1–3^, such as agricultural pest control and soil and water conservation^4–7^. The diversity of wild plants in agroecosystems is an important part of global biodiversity^8^. It is noteworthy that herbicides have changed the species composition of weed communities, the number of plants sensitive to herbicides has decreased, and the number of plants resistant to herbicides has increased in farmlands^9,10^. Herbicides are widely used in farmlands worldwide to increase crop yield and farm labour efficiency^11^, so the effect on the wild plant community is unlikely to cease as herbicide use in agricultural production^12,13^. However, the full amount of the applied herbicides does not reach the targets; a large proportion of herbicides drift and run off from sprayed fields towards adjacent non-target areas^14^.

The ecological risks of herbicides, especially low concentrations or sublethal doses of herbicides, to non-target wild plant communities in agroecosystems has not been adequately assessed. In contrast, many studies have focused on the effect of herbicides on the biomass, reproduction, and progeny of individual plants^15–18^.While these studies revealed that herbicides damage the ecological adaptability of plants, a knowledge gap remains between the study of individual plants and the actual goal of plant community protection^19^. Fortunately, much attention has been paid to the effects of herbicides on the non-target wild plant community of non-crop habitats, especially field margins in agricultural landscapes^20–22^. Fallow fields, which are common non-crop habitats in agroecosystems, provide shelter for wild plants and animals near agricultural fields^1^ and are often affected by herbicide drift from fields. However, there have been very few studies on the effects of herbicides on the plant communities in fallow fields, which is the basis for models predicting herbicide risk^23^; thus, the ecological risk of herbicides may be underestimated^24^. An understanding how herbicides affect the diversity and functional composition of non-target plant communities in fallow fields, is needed to provide baseline information for biodiversity conservation and deliver wise management decisions for agricultural landscapes^25,26^.

With the rapid development of China’s economy, the demand for cereal crops has been increasing; moreover, a large number of rural agricultural workers have moved to cities. Thus, herbicides have been widely used to compensate for the lack of labour that was used to perform weeding tasks in China. In response to the problem of agricultural pollution, the “Action Plan for Zero Growth in Pesticide Use by 2020” was issued by the Chinese government in 2015 (http://jiuban.moa.gov.cn/zwllm/tzgg/tz/201503/t20150318_4444765.htm), and herbicides, as the most commonly used pesticide, are the focus of the initiative. A full understanding of the ecological impact of herbicides is the basis for the formulation of scientific management policies. Previous studies on the ecological risks of herbicides were mostly carried out in Western Europe and North America^19,21^. However, ecological risk assessments of herbicides depends on the susceptibility of native plants, crop management operations and natural environmental factors; therefore, there is a need for more work on the ecological risks of herbicides in China. In addition, China has a large amount of arable land lying fallow, which has been affected by herbicides from the surrounding farmlands because of the loss of the rural labour force in China.

Accordingly, a 3-year field study with a randomized block design was performed to investigate the effect of two herbicides on the plant community of a fallow field in northern China. Atrazine and tribenuron-methyl were among the most widely used herbicides in field grain crops in China and are representative of a range of chemical groups with different modes of action^27,28^. The major objectives were to (i) evaluate the effects of atrazine and tribenuron-methyl on the plant community in the fallow field, (ii) determine if increased dose exposure of the plant community increases the impact on community diversity and functional composition, and (iii) determine whether herbicides have cumulative effects on plant communities over time, i.e., years of exposure.

## Materials and methods

### Site description

This experiment was carried out at the experimental station of Chinese Research Academy of Environmental Sciences. The location was the town of Zhaoquanying, Shunyi district, Beijing (115.7°–117.4°E, 39.4°–41.6°N; 20–60 MASL), in China’s Huang-Huai-Hai plain. Beijing has a semi-humid monsoon climate with distinct seasons. The mean temperature is −4 °C in January and 26 °C in July and August. The annual surface evaporation is 1800 mm, and the mean annual rainfall is 655 mm. The precipitation is unevenly distributed, with more than 80% occurring from June-August^29^. The main grain crops in Shunyi district are wheat and corn^30^. The study site was a fallow fields (approximately 0.16 ha) surrounded by other fallow fields and did not encounter any herbicide drift during the experiment. The soil of the study site was fluvo-aquic. The pH of the soil was 7.5, and the soil organic carbon content was 6.93 g·kg^−1^. Before the experiment, corn and rape were planted without pesticides for three years, and the dominant plant species were *Digitaria sanguinalis*, *Amaranthus retroflexus*, and *Abutilon theophrasti*. In April 2014, the ground was ploughed with machinery after artificial removal of plant debris from the surface.

### Herbicide selection

Two herbicides were selected for use in the experiments. Atrazine (2-chloro-4-ethylamino-6-isopropylamino-1,3,5-triazine) binds to the plastoquinone binding site in the photosynthetic electron transport system and inhibits photosynthesis^31^. Atrazine is an atrazine herbicide used for soil and leaf treatments and is persistent in the environment^32^. The 100% atrazine dose recommended for northern China on the product label is 1200 g a.i. ha^−1^ (GREEN LAND, Shandong Shengbang Greenland Chemical Co., Ltd., Jinan, China). Tribenuron-methyl (methyl 2-[4-methoxy-6-methyl-1,3,5-triazin-2-yl (methyl) carbamoylsulfamoyl] benzoate) inhibits acetolactate synthase, a key enzyme in the biosynthesis of branched chain amino acids^33^. It is rapidly absorbed by plant leaves^34^. 100% tribenuron-methyl dose as recommended for northern China on the product label is 22.5 g a.i. ha^−1^ (QCC, Shandong Qiaochang Chemical Co., Ltd., Binzhou, China).

### Experimental design

In this experiment, we applied three concentrations for each of the two herbicides and one blank control in a randomized complete block design with four replications. There were 28 plots in total, and we randomly assigned one of the concentrations of herbicides to each plot within each block. The 3 m by 5 m plots were separated from each other by 3 m on all sides to eliminate interactions between the plots. Three concentrations of herbicides were tested: 100%, 50%, and 25% of the recommended field application concentrations (RFAC) (where 100% is full strength). On June 18th or 19th of each year (from 2014 to 2016), each plot within each block was sprayed with 1200 g a.i. ha^−1^ (100% RFAC), 600 g a.i. ha^−1^ (50% RFAC), or 300 g a.i. ha^−1^ (25% RFAC) of atrazine; or 22.5 g a.i. ha^−1^ (100% RFAC), 11.25 g a.i. ha^−1^ (50% RFAC), or 5.63 g a.i. ha^−1^ (25% RFAC) of tribenuron-methyl using a manual sprayer with a cone-shaped nozzle (NS-5, Shanghai Worth Garden Co., Ltd., Shanghai, China). The blank control was sprayed with water. No additional surfactants or other adjuvants were used in the treatments. The herbicides were applied at the same time as they would have been applied to the local summer corn crop. Summer corn is a major crop in China’s Huang-Huai-Hai Plain, which is one of China’s two dominant corn production areas. Plastic film and bamboo poles were used to build a 1.5-m-high separation barrier in each plot to prevent the herbicides from floating around for 48 h after the herbicide application. The area between the plots was mowed 2–3 times a year.

### Sampling and measurements

Plant species composition and community diversity were assessed from 2014 to 2016. Plant samples were taken four times a year during the growing season in early June (before the annual herbicide treatment) and in early August, early September, and early October. Data recorded in early June of 2014 served as the background observation value of plants in this study. Three 0.5 m × 0.5 m sample quadrats were randomly distributed in each plot to record the species and number of plants. Each quadrat was no less than 0.5 m away from the plot edge. The number of shoots for each plant species was counted in each sample quadrat.

The plant species were identified at the species level whenever possible, and some species, including *Setaria* spp. and *Bidens* spp., were identified at the genus level according to the websites of the “Flora of China” (http://foc.iplant.cn/) and the Chinese Virtual Herbarium (http://www.cvh.ac.cn/). The mean abundance of each plant species was calculated in four repeated plots of the same treatment for each observation. The total number of species was estimated by all the species rooted within each of the quadrats in the plots of the same treatment at each of the four samples taken each year. For each species, the mean relative frequencies were estimated by recording the presence or absence of all the species rooted in each quadrat of the four repeated plots of the same treatment at the four sample times each year over three years. The Margalef species richness index, Shannon’s diversity index, and Pielou evenness were used as measures of species diversity. The Margalef species richness index was calculated as follows: R = (S − 1)/InN. Shannon’s diversity index was calculated as follows: ${\rm{H}}=-{\sum }_{{\rm{i}}=1}^{{\rm{s}}}{\rm{PilnPi}}$. Pielou evenness was calculated as follows: E = H/InS, where S is the total number of species per quadrat, N is the total number of individuals observed per quadrat, and Pi is the proportion of individuals of species i in the total number of individuals in aquadrat^35^.

###  Statistical analysis

We combined RLQ and fourth-corner analyses to test the covariation between the herbicide treatment variables (R table) and plant species traits (Q table), constrained by the mean abundance of each plant species (L table)^36,37^. Five functional traits of plant ecological performance were chosen on the basis of the potential effect of herbicide type, herbicide dose, and time (year and month) of the application. Trait information for the plant species observed in this study was obtained from the website “Flora of China”. The plants that could not be determined at the species level were excluded from the combined RLQ and the fourth-corner analyses. The five functional traits were life form (annual, biennial, and perennial), number of cotyledons (monocotyledon and dicotyledon), flowering onset (month), seed length (cm), and plant height (cm). We used 49,999 permutations in all the randomization procedures and a false discovery rate method to adjust the P values for the multiple tests used in the fourth-corner analysis and the combination of the RLQ and fourth-corner analyses.

An independent t test was used to analyse the difference in the mean frequency of each plant species between each herbicide-treated plot and the blank control. Repeated measures MANOVA was used to analyse the main effects of observation time, herbicide type, herbicide dose, and their interaction on the Margalef species richness index, Shannon’s diversity index, and Pielou evenness^38,39^. If the data met the assumptions of Mauchly’s test, the results of the internal effect table were used; if the data did not meet the assumptions of Mauchly’s test, the results of the MANOVA were used, and Pillai’s trace was used as the multivariate test statistic^40^. One-way ANOVA was used to analyse the differences in the Margalef species richness index, Shannon’s diversity index and Pielou evenness between the herbicide doses. MANOVA and ANOVA were employed to test the differences between the means from the experiments. The data are shown as the mean ± standard deviation.

## Results

### Effects of herbicides

**Figure 1:**
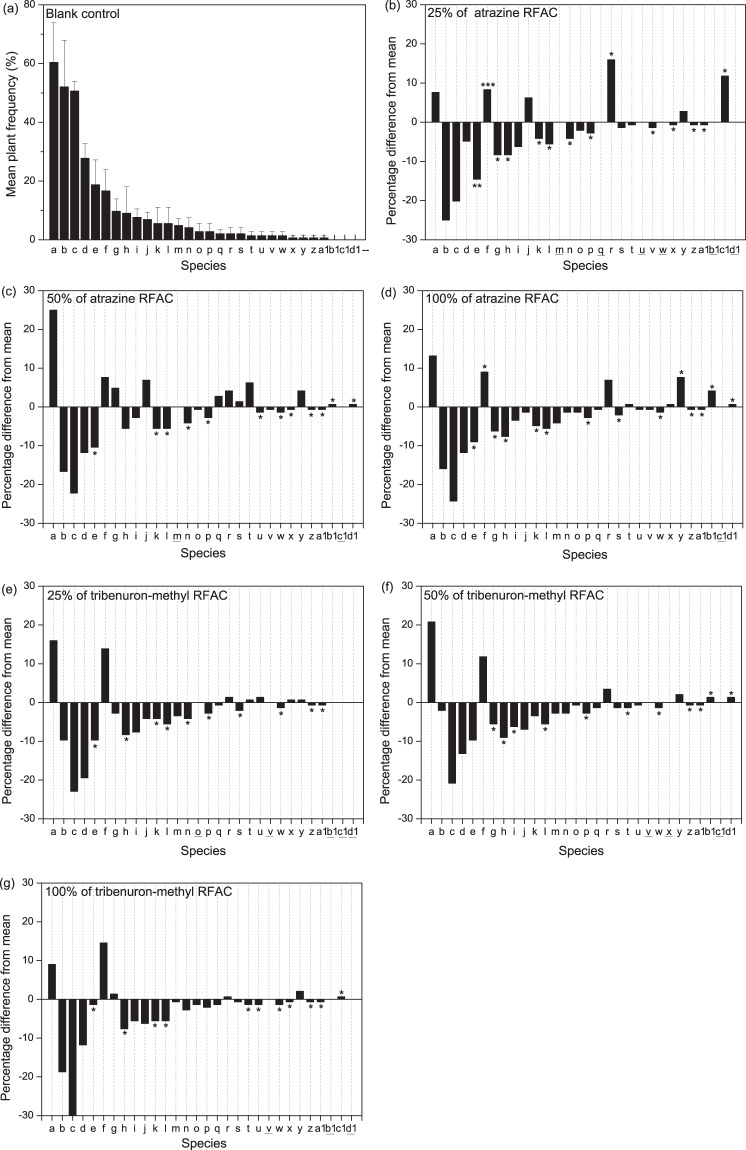
Relative frequency of occurrence of 31 plants across the quadrats: (a) mean frequency of plant sample without herbicide; (**b**–**g**) the frequency of plant sample treated with herbicide compared with mean frequency of plant sample without herbicide. Note: data are the percentage of all quadrat cells in which the species was recorded in June, August, September, and October in 2014, 2015 and 2016. Here, and in Fig. 2, species codes are: a:*Digitariasanguinalis;* b:*Amaranthusretroflexus*; c: *Chenopodiumalbum*; d: *Abutilon theophrasti*; e:*Setaria* spp.; f:*Calystegia hederacea*; g:*Setariaviridis*; h:*Acalyphaaustralis*; i: *Metaplexis japonica*; j: *Bidens* spp.; k:*Luffa aegyptiaca*; l: *Xanthium sibiricum*; m:*Brassica rapa var. oleifera*; n: *Portulacaoleracea*; o-*Ipomoea purpurea*; p:*Erigeron canadensis*; q:*Ixerispolycephala*; r:*Humulusscandens*; s:*Lactucaindica*; t: *Ambrosia artemisiifolia*; u: *Glycine soja*; v-*Eleusineindica*; w- *Picris japonica*; x: *Solanum americanum*; y: *Echinochloacrusgalli*; z: *Cyperusmicroiria*; a1:*Rorippaglobosa*; b1: *Ipomoea nil*; c1:*Cirsiumarvense var. integrifolium*; d1:*Capsella bursa-pastoris*. The horizontal line under a letter indicates that the relative frequency of a species in this treatment did not change that of the blank control. *Indicates the significance of the difference between the frequency of different treatments and the frequency of blank treatments for each species according to the independent-sample ttest. *P < 0.05, **P < 0.01, and ***P < 0.001.

Both atrazine and tribenuron-methyl changed the species composition of the plant community. The total number of plant species in the plots treated with the herbicide was lower than that in the blank control plots (Table 1). In the blank control, the species belong to 26 genera; however, in the plots treated with 25%, 50%, and 100% of the RFAC of atrazine, the species belong to 20, 18, and 21 genera, and in the plots treated with 25%, 50%, and 100% of the RFAC of tribenuron-methyl, the species belong to 18, 19, and 19 genera, respectively. Moreover, both herbicides significantly altered the relative frequency of some plants but had no significant effect on the species with relatively high frequencies in the blank control, including *Digitaria sanguinalis*, *Amaranthus retroflexus*, *Chenopodium album*, and *Abutilon theophrasti* (Fig. 1). The number of plant species whose relative frequency was inhibited by herbicides was higher than the number of plant species whose relative frequency was increased by herbicides (Fig. 1). However, based on the fourth-corner analysis, herbicide type had no significant effect on plants with different life forms, different numbers of cotyledons, different initial flowering months, or different plant heights and seed sizes.

**Table 1:** Total number of plant species of different treatments for each year.

Treatment	Year			Total
	2014	2015	2016	
**Blank control**	13	14	24	27
**25% of atrazine**	11	15	15	21
**50% of atrazine**	13	14	16	20
**100% of atrazine**	14	14	15	23
**25% of tribenuron-methyl**	12	12	10	19
**50% of tribenuron-methyl**	11	11	16	21
**100% of tribenuron-methyl**	13	13	11	20

**Figure 2:**
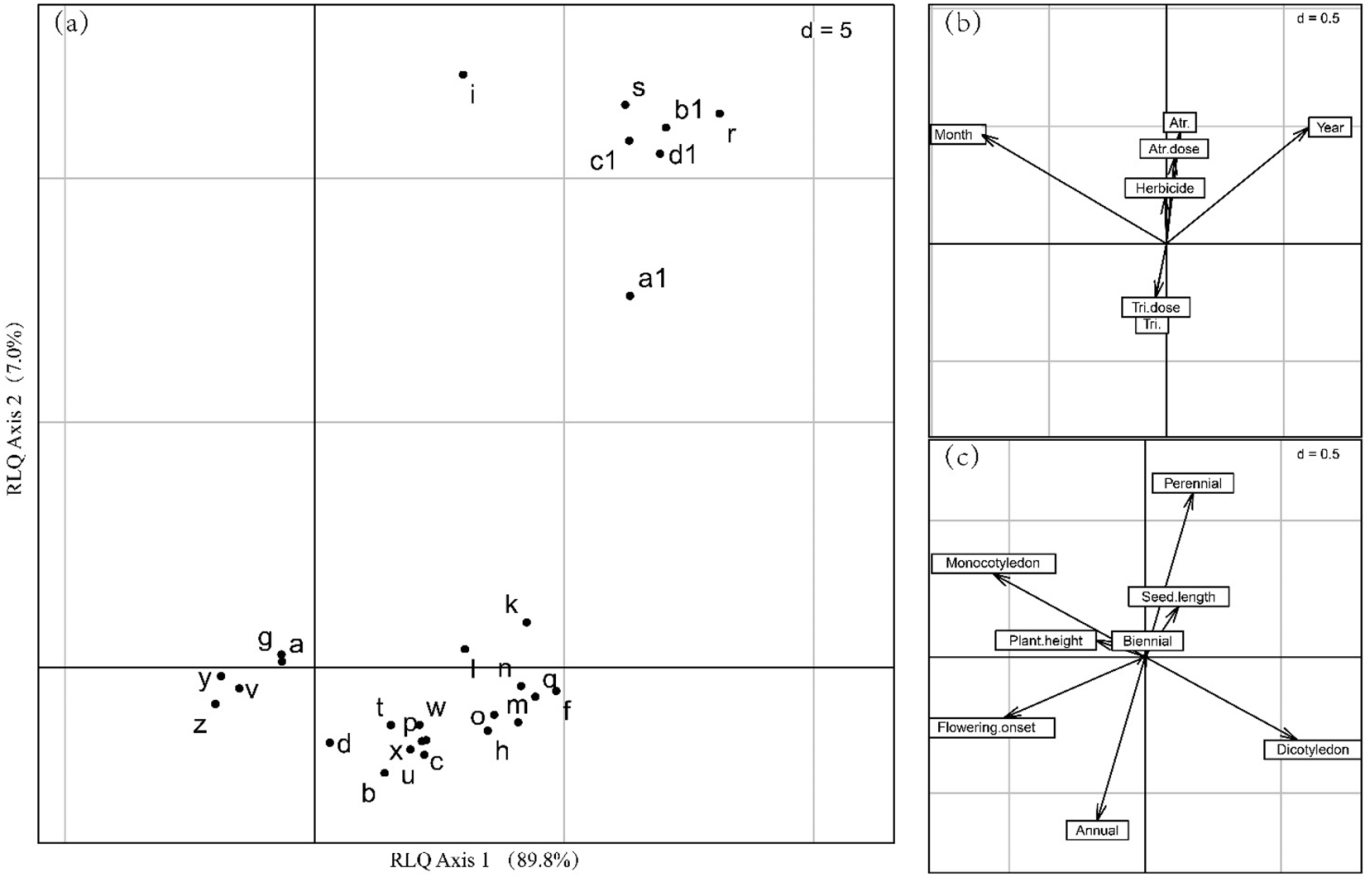
Results of the first two axes of the RLQ analysis: (**a**) scores of species, (**b**) coefficients for environmental variables, and (**c**) traits. The values of *d* give the grid size. Atr.: atrazine; Tri.: tribenuron-methyl.

The effects of atrazine and tribenuron-methyl on plant community composition were not consistent. The total number of species in plots treated with the RFAC (in 2014) and 25% of the RFAC (in 2015) of atrazine was higher than that in the blank control plots; however, the total number of species in the plots treated with tribenuron-methyl was not higher than that in the blank control plots (Table 1). The relative frequency was significantly changed for more plants by atrazine (Fig. 1b−d) than by tribenuron-methyl (Fig. 1e−g). Moreover, the upper (positive) part of the second RLQ axis identifies perennial species (i: *Metaplexis japonica*, s: *Lactucaindica*, c1: *Cirsiumarvense var. integrifolium*, Fig. 2a) with relatively long seeds (seed length) (Fig. 2c), which were mostly found in the herbicide-treated plots, particularly in the atrazine-treated plots (Fig. 2b). However, the lower (positive) part of the second RLQ axis identifies annual species (v: *Eleusineindica*, z*: Cyperus microiria*, Fig. 2a), which were mostly found in the tribenuron-methyl-treated plots (Fig. 2b).

**Figure 3:**
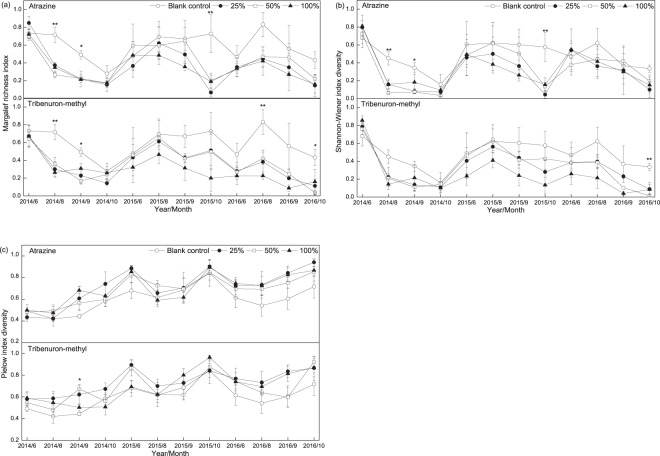
Margalef species richness index (**a**), Shannon’s diversity index (**b**), and Pielow evenness (**c**) of the plant community treated with atrazine or tribenuron methyl for three years. Note: * shows the difference in the plant community diversity index in the different herbicide dosages at the same time according to one-way ANOVA. *P < 0.05, **P < 0.01, and ***P < 0.001.

Herbicides reduced the plant community diversity in the fallow field, but there was no significant difference in the effects of atrazine or tribenuron-methyl on the species diversity indices of the plant community (Table 2). Compared with the blank control, atrazine significantly reduced the Margalef species richness index in August 2014 (*P* < 0.01) and October 2015 (*P* < 0.01) and Shannon’s diversity index in August 2014 (*P* < 0.01) and October 2015 (*P* < 0.01); tribenuron-methyl significantly reduced Margalef species richness index in August 2014 and 2015 (*P* < 0.01) and Shannon’s diversity index in October 2016 (*P* < 0.01) (Fig. 3a,b).

**Table 2:** Repeated measurement MANOVA for testing for time, herbicide type, dose and their interactions on the diversity index of the plant community.

Index	Source of variation	d. f.	Pillai’s Trace	Mean	*F* ratio	*P* value
			Value (Error d. f.)	Square		
Margalef	**T**	11	0.971(14)	—	42.167	<0.001***
species	**HT**	1	—	0.066	0.493	0.489
richness	**HD**	3	—	1.378	10.229	<0.001***
index	**T×HT**	11	0.466 (14)	—	1.111	0.419
	**T×HD**	11	1.341(48)	—	1.176	0.299
	**T×HT×HD**	33	0.867(48)	—	0.591	0.943
Shannon-	**T**	11	0.986(14)	—	89.088	<0.001***
Wiener	**HT**	1	—	0.036	0.301	0.588
index	**HD**	3	—	0.649	5.479	0.005**
diversity	**T×HT**	11	0.384(14)	—	0.792	0.646
	**T×HD**	11	1.459(48)	—	1.378	0.153
	**T×HT×HD**	33	0.606(48)	—	0.368	0.998
Pielouinde	**T**	11	0.945(14)	—	21.703	<0.001***
x diversity	**HT**	1	—	<0.001	0.003	0.953
	**HD**	3	—	0.266	5.744	0.004**
	**T×HT**	11	0.352(14)	—	0.691	0.728
	**T×HD**	11	1.401(48)	—	1.274	0.219
	**T×HT×HD**	33	0.885(48)	—	0.609	0.932

T: time, HT: herbicide type, HD: herbicide dose. d.f.: degrees of freedom; ns: not significant. *P < 0.05, **P < 0.01, and ***P < 0.001.

### Effects of the herbicide dose

The MANOVA demonstrated that the herbicide dose had a significant impact on the Margalef species richness index (*P* < 0.001), Shannon’s diversity index (*P* < 0.01), and Pielou evenness (*P* < 0.01) of the plant community (Table 2). However, the effects of the herbicide doses that were less than the RFAC on the plant community composition and community diversity of the fallow field were not lower than the effects of the RFAC of the herbicides. Compared with the blank control, the inhibition by atrazine or tribenuron-methyl on the total number of species in the plots did not increase with the increase in dose (Table 1). Furthermore, the number of species with significant changes in relative frequency was 14, 12, and 14 in the plots treated with 25%, 50%, or 100% of the RFAC of atrazine, respectively (Fig. 1b−d). In addition, Shannon’s diversity index was significantly reduced in the herbicide-treated plots compared with that in the blank control plots in August 2014, October 2015 (atrazine), and October 2016 (tribenuron-methyl), while there was no significant difference in the level of inhibition by 25%, 50%, and 100% of the RFAC of atrazine or tribenuron-methyl on Shannon’s diversity index according to the one-way ANOVA (Fig. 3b). There was also no significant difference in the inhibition caused by atrazine and tribenuron-methyl (at 25%, 50%, and 100% of the RFAC) according to the Margalef species richness index of the plant community in August 2014, September 2014, and October 2015 for atrazine, and August 2014 and 2016 for tribenuron-methyl (Fig. 3a).

Herbicide doses that were lower than the RFAC had an even greater impact on community diversity than the RFACs of the herbicides. For example, compared with the blank control, the RFAC of atrazine (*P* > 0.05) did not have a significant effect on Shannon’s diversity index of the plant community, whereas 25% (*P* < 0.01) and 50% (*P* < 0.01) of the RFAC of atrazine significantly reduced Shannon’s diversity index in September 2014 according to the one-way ANOVA (Fig. 3b). The effects of different concentrations of tribenuron-methyl on the Margalef species richness index in September 2014 and October 2016 were similar to those of atrazine on Shannon’s diversity index in September 2014 (Fig. 3a).

However, according to the fourth-corner analysis, herbicide dose had no significant effect on plants with different life forms, different numbers of cotyledons, different initial flowering months, or different plant heights and seed sizes. The plots with relatively high concentrations of atrazine were found in the upper (positive) part of the second RLQ axis. These plots were characterized by perennial plants with longer seeds (Fig. 2b,c). Pots with relatively high concentrations of tribenuron-methyl were found in the lower (positive) part of the second RLQ axis. These plots were characterized by annual plants with late flowering onset (Fig. 2b,c).

### Effect of time

As the number of years of herbicide application increased, the number of plant species decreased and the inhibitory effect of the herbicides was enhanced. For example, the total number of plant species in the plots treated with 25% and 100% of the RFAC of tribenuron-methyl was not higher than that in the blank control plot in 2014, 2015, or 2016. Indeed, the total number of plant species in the plots treated with 25% or 100% of the RFAC of tribenuron-methyl in 2016 was lower than that in the same plots in 2014 and 2015 (Table 1). Moreover, time significantly affected the Margalef species richness index (*P* < 0.001), Shannon’s diversity index (*P* < 0.001), and Pielou evenness (*P* < 0.001) of the plant community in the fallow field (Table 2). The variation in the diversity index between the plots treated with the different herbicides and the blank control was not consistent during the different months of the plant growing season throughout the year (Fig. 3). The effects of atrazine or tribenuron-methyl on plant community diversity did not change significantly after three years of continuous herbicide application (Fig. 3). The interactions (time × herbicide type, time × herbicide dose, and time × herbicide type × dose) had no significant effect on the Margalef species richness index, Shannon’s diversity index, or Pielou evenness of the plant community (Table 2).

However, the observation time had no significant effect on the functional composition of plants. The right part of the first RLQ axis highlights the trait attributes (dicotyledonous, longer seeds, and perennial life cycle) associated with the increase in observed years (Fig. 2b,c). The left (negative) part of the first RLQ axis highlights the trait attributes (monocotyledonous and later flowering onset) associated with the later growing season (September and October) (Fig. 2b,c). The year and month in which observations were recorded had no significant effect on the presence of plants with different life forms, different numbers of cotyledons, different initial flowering months, or different heights or seed sizes based on the fourth-corner analysis.

## Discussion

Plant communities in field edges, fallow fields, and other semi-natural habitats of agricultural landscapes may be at significant ecotoxicological risk from herbicides applied to nearby crop fields^21^. In agricultural landscapes, field margins, fallow fields, and other semi-natural habitats are often the only remaining habitat for wild plant species and support diverse plant communities that help sustain pollinators, predators, and beneficial arthropods^21,41^. Previous studies have indicated that herbicides, even at low concentrations, adversely affect plant communities, causing a decline in forb cover and reduced the flowering of key species^21^ and a reduction in the frequencies of certain species^41^. Our data on plant species diversity and community composition of a fallow field support these findings. However, some studies have suggested that herbicides may not reduce the diversity of plant communities in agricultural landscapes^42,43^.

The important result of our study is that atrazine and tribenuron-methyl altered the species composition and reduced the diversity of the plant communities in the fallow field in no more than three years. Our results were partly due to the species-specific sensitivity of plants to these selective herbicides^44^. Atrazine had little effect on perennials, while tribenuron-methyl had little effect on monocotyledons (Fig. 2) because of different chemical properties, weeding mechanisms, and weed control targets^31,45^. Herbicide types were one of the important factors affecting the diversity of weed communities^42,46^. Furthermore, the response of plant communities to herbicides is related to community characteristics^47,48^. The succession of the vegetation with fallow age also revealed a gradient of plant strategies^1,49^. In this study, the fallow field was in an early fallow stages immediately following agricultural disturbances. The communities in early fallow stages are often dominated by opportunistic ruderal species with fast growth and an annual life cycle^1^. The herbicides, particularly atrazine, killed or inhibited annuals, which may be one of the reasons for annuals appearing more frequently in the blank control plots and perennials appearing more frequently in the herbicide treated plots. In aggregate, the herbicides altered the plant species composition and reduced plant community diversity by reducing the number of species in the fallow field. Although species richness is not necessarily the most suitable indicator of healthy non-crop habitats in adjacent farmlands because some species are known to respond positively to disturbances^20^, changes in the number of species will inevitably affect the plant interactions in a plant community.

Herbicides may not be an important factor in changing the functional composition of plant communities in the short term, but the long-term cumulative effects of herbicides on the functional composition and structure of plant communities in agricultural ecosystems need to be taken seriously^50^. In this study, although atrazine and tribenuron-methyl both reduced the number of plant species, they did not significantly change the functional composition of the plant communities within three years. Previous studies have shown that fertilizer drift appeared to have a much stronger effect than herbicide drift on the plant trait composition in field margin strips^20^; however, a significant interaction between herbicides and other agrochemicals shifted the functional structure of communities over the course of 11 years^50^. Although plant functional trait analysis could better reveal the value of non-food plant resources to food production and biodiversity in agroecosystems^4,51^, studies on the effects of herbicides on plant functional groups in agroecosystems are limited.

It is worth noting that contrary to our assumptions, herbicide doses less than the RFAC had a similar effect to the RFAC of the herbicides on the total number of species (Table 1) and their relative frequencies (Fig. 1) and the diversity index (Fig. 3), as with the findings of other studies on forb cover at field edges^21^. Our data also showed that herbicide doses less than the RFACs had an even greater impact on the diversity index than the RFACs of the herbicides. These results may be explained by the effects of herbicides on the growth of individual plants. Herbicide doses less than the RFAC could damage plant growth^52,53^ and reduce seed production^44^, which could directly change the soil seed bank input. From another perspective, we could explain the results by the effects of the herbicides on the plant offspring. Sublethal herbicides have been shown to affect the germination and seedling growth of the F1 generation of plants, although species-specific responses were not consistent^18^. The effects of sublethal herbicides on plants could change the development of the plant community; thus, plant community diversity and species composition may be changed. With this in mind, more attention should be paid to the prevention and control of the ecological risks of low concentrations or sublethal doses of herbicides in agricultural landscapes, which are often caused by herbicide drift and runoff from sprayed fields into adjacent non-target areas^44,54^. A certain width of herbicide-free isolated areas could be maintained between farmlands and non-target areas to controls ecological risks^55^.

Herbicides are applied to farmland at least once a year and are used in most fields for many years, so the time factor has often been considered in research on the influence of herbicides on plant diversity in an agricultural landscapes^21^. In this study, the effects of herbicides on plant community diversity during different months of the growing season in a year were inconsistent (Fig. 3). This may be because different plants have different growth cycles and different species have different response times to herbicides^56^. Thus, observations made during different months of the year should be cautiously compared in studies on the effect of herbicides on plant communities. On the other hand, previous studies (as well as this study) have shown that the effects of herbicide application over many years on plant community diversity were irregular^57^. The effect of herbicides on the development of species communities was evident over time^52^. However, abiotic conditions and other unmeasured deterministic or stochastic processes, which could be driving observed plant patterns, may affect the amount of time it takes for herbicides to alter plant communitiesy^26^.

These findings must be interpreted with caution for several reasons. Our experiments only tested two herbicides commonly used in China as well as plant communities in a 3-year field study with a randomized block design; therefore, they represent just one sample of semi-natural habitats in the agricultural landscape of China. We used only two of the commonly utilized herbicides from a long list of herbicides that are frequently applied to Chinese farmland. Moreover, we did not consider the effects of herbicide application time after three years on the plant community in the agricultural landscape, which may result in an underestimation of the ecological risks arising from herbicides applied to wild plants in fallow fields. Thus, studies on the ecological risks of herbicides should be focused on the differences and similarities between different categories of herbicides to better guide the prevention of ecological risks of herbicides to the non-target plant communities in actual agricultural production areas. Field experiments and observations should continue to be carried; increased observation periods, different semi-natural habitats in agricultural landscapes, and an increased number of herbicides should be used to more deeply reveal the effects of herbicides on non-target wild plant communities in agricultural landscapes.
